# T110. FIRST EPISODE PSYCHOTIC PATIENTS WITH A HISTORY OF FREQUENT CANNABIS USE EXPRESS MORE POSITIVE SYMPTOMS AT ILLNESS ONSET THAN THOSE WHO NEVER USED CANNABIS

**DOI:** 10.1093/schbul/sby016.386

**Published:** 2018-04-01

**Authors:** Diego Quattrone, Charlotte Gayer-Anderson, Laura Ferraro, Giada Tripoli, EUGEI group, Evangelos Vassos, Pak Sham, Jim van Os, Craig Morgan, Cathryn Lewis, Ulrich Reininghaus, Robin Murray, Marta Di Forti

**Affiliations:** 1 Institute of Psychiatry, Psychology & Neuroscience, King’s College London; 2 University of Palermo; 3 European Network; 4 The University of Hong Kong; 5 Maastricht University

## Abstract

**Background:**

Robust evidence has demonstrated that cannabis use increases the risk to develop psychotic disorders. However, a limited number of studies have investigated if and how cannabis use influences psychopathology profiles at first episode psychosis (FEP).

Based on the evidence that dopamine dysfunction contributes to explain positive symptoms in psychosis, and that the main cannabis’ psychoactive component, Δ9-Tetrahydrocannabinol (THC), modulates the dopamine system, we hypothesise that: 1) positive symptoms at FEP are more common among psychotic patients who used cannabis compared with never users; 2) this association is a dose-response relationship.

**Methods:**

We analyzed a sample of 1130 FEP patients as part of the EUGEI study, recruited across six countries. The MRC Socio-demographic Schedule was used to collect sociodemographic information. Psychopathology was assessed with the OPerational CRITeria (OPCRIT), and symptom items were analyzed using Mplus to estimate a multidimensional model of psychosis. The Cannabis Experience Questionnaire modified version (CEQmv) was administered to collect information on cannabis, and different patterns of use were computed based on frequency of consumption and type of cannabis, as a proxy of exposure to THC.

**Results:**

The lifetime rate of cannabis use was 63%. Fifty-five percent of cannabis users consumed mostly high-potency cannabis, and 46% showed a daily frequency. Mixed-effects linear regression revealed that frequency of cannabis use was associated with the positive symptom dimension score. Daily users of high-potency cannabis presented with the strongest association (Β=0.19, 95%CI=0.02–0.38), even after gender, age, ethnicity, other drug use, and study site were controlled for.

**Discussion:**

Our results show that patients with a history of daily use of high potency cannabis express more positive symptoms at psychosis onset, even after taking into account other substance use and relevant sociodemographic factors.

